# Tea Polyphenols and Their Roles in Cancer Prevention and Chemotherapy

**DOI:** 10.3390/ijms9071196

**Published:** 2008-07-12

**Authors:** Di Chen, Q. Ping Dou

**Affiliations:** The Prevention Program, Barbara Ann Karmanos Cancer Institute, and Department of Pathology, School of Medicine, Wayne State University, Detroit, Michigan, USA

**Keywords:** tea polyphenols, cancer prevention, chemotherapy

## Abstract

Many plant-derived, dietary polyphenols have been studied for their chemopreventive and chemotherapeutic properties against human cancers, including green tea polyphenols, genistein (found in soy), apigenin (celery, parsley), luteolin (broccoli), quercetin (onions), kaempferol (broccoli, grapefruits), curcumin (turmeric), etc. The more we understand their involved molecular mechanisms and cellular targets, the better we could utilize these “natural gifts” for the prevention and treatment of human cancer. Furthermore, better understanding of their structure-activity relationships will guide synthesis of analog compounds with improved bio-availability, stability, potency and specificity. This review focuses on green tea polyphenols and seeks to summarize several reported biological effects of tea polyphenols in human cancer systems, highlight the molecular targets and pathways identified, and discuss the role of tea polyphenols in the prevention and treatment of human cancer. The review also briefly describes several other dietary polyphenols and their biological effects on cancer prevention and chemotherapy.

## 1. Introduction

It is estimated by the American Cancer Society that in 2007, there will have been more than 12.3 million new cancer cases and 7.6 million deaths from cancers worldwide [1]. How to decrease cancer incidence and mortality has been a major challenge in this endeavor. The growing amount of evidence from studies in epidemiology, cell cultures and animal tumor models demonstrates that a large number of natural compounds from the diet could lower cancer risk and some of them could sensitize tumor cells in anti-cancer therapies [2–5]. For cancer prevention and chemotherapy, plant-derived natural compounds are an invaluable treasure and worthy to be further explored. In this review we summarize the effects of some well studied natural compounds, with green tea polyphenols as a focus, against cancers and their potential molecular targets.

## 2. Natural Compounds and Their Molecular Targets for Cancer Prevention and Treatment

### 2.1. Tea Polyphenols

The history of tea began in ancient China over 5,000 years ago. Tea, of all varieties, is the most widely consumed beverage in the world today, and is consumed by 1/3 of the world'spopulation. Green tea, black tea, and oolong tea are all derived from the *Camellia sinensis* plant. Of all the teas consumed in the world, green tea is well studied for their health benefits [6]. It is generally agreed that the cancer chemopreventive effects of green tea are mediated by its abundant polyphenol, epigallocatechin gallate [(-)-EGCG].

#### 2.1.1. (-)-EGCG Inhibit Proteasome Activity in Tumor Cells

It has been suggested that proteasome activity is essential for tumor cell proliferation and drug resistance development. Therefore, the proteasome-mediated degradation pathway has been considered an important target for cancer prevention and therapy. The proteasome inhibitor Bortezomib (Velcade, PS-341) has been used in clinical trials and its antitumor activity has been reported in a variety of tumor models [7–9]. The ubiquitin/proteasome system controls the turn-over of critical regulatory proteins involved in several cellular processes such as cell cycle and apoptosis [10, 11]. Under normal conditions, the lysosomal pathway degrades extracellular proteins imported into the cell by endocytosis or pinocytosis, whereas the proteasome controls degradation of intracellular proteins [10, 12]. The eukaryotic proteasome contains at least three known catalytic activities: chymotrypsin-like, trypsin-like, and caspase-like or peptidyl-glutamyl peptide-hydrolyzing (PGPH)-like activities [13]. Our laboratory and others, have reported that inhibition of the proteasome chymotrypsin-like activity is associated with induction of apoptosis in tumor cells [14, 15]. We reported that (-)-EGCG potently and specifically inhibited the chymotrypsin-like activity of the proteasome *in vitro*(IC_50_ = 86–194 nM) and *in vivo* (1–10 μM) at the concentrations found in the serum of green tea drinkers and induced tumor cell growth arrest in G_1_ phase of the cell cycle [16]. We also reported for the first time, that an ester bond within (-)-EGCG played a critical role in its inhibitory activity of the proteasome [16]. We found that synthetic (-)-EGCG amides and (-)-EGCG analogs, with modifications in the A-ring, C-ring or ester bond, inhibited the chymotrypsin-like activity of purified 20S proteasome with altered potencies, induced growth arrest in the G_1_ phase of the cell cycle in leukemia Jurkat T cells, and suppressed colony formation of human prostate cancer LNCaP cells. However, these EGCG analogs caused little or no proteasome inhibition in normal or non-transformed cells [17].

(-)-EGCG remains the most potent polyphenol in green tea, but one of the limitations of (-)-EGCG is its instability in neutral or alkaline conditions (*i.e.* physiologic pH). In an effort to discover more stable polyphenol proteasome inhibitors, we synthesized several (-)-EGCG analogs with −OH groups eliminated from the B- and/or D-rings. In addition, we also synthesized their putative prodrugs with −OH groups protected by acetate that can be removed by cellular cytosolic esterases. We first examined the structure-activity relationship of these unprotected and protected compounds with respect to their proteasome inhibitory potentials. We found that decreasing −OH groups from either the B- or D-ring leads to diminish proteasome-inhibitory activity *in vitro*. However, in cultured tumor cells, the protected analogs were able to inhibit the proteasomal chymotrypsin-like activity by as much as 97% [18]. Furthermore, we found that the protected analogs exhibited greater potency compared to (-)-EGCG regarding inhibited proliferation and transforming activity and induction of apoptosis in human leukemic, prostate, breast, and simian virus 40-transformed cells [19, 20]. The protected analogs were non-toxic to human normal and non-transformed cells [19, 20].

#### 2.1.2. (-)-EGCG Protects DNA from Methylation

In the process of carcinogenesis, a carcinogen may cause changes in gene functions and/or in gene constructions. Epigenetic silencing by hypermethylation of tumor suppressor or DNA repair-related genes occurs more frequently during the early stages of the neoplastic process and may result in carcinogenesis in cells [21].

It has been reported that silencing of the O^6^-methylguanine-DNA methyltransferase gene (*MGMT*) results in cells with the ability to acquire a specific type of genetic mutation in p53, and subsequently, an inability to repair DNA guanosine adducts [22].

Fang *et al*. [23] reported that (-)-EGCG could inhibit the activity of DNA methyltransferase (DNMT), resulting in CpG demethylation and reactivation of methylation-silenced genes in human esophageal cancer KYSE 510 cells. In this study, KYSE 510 cells treated with 5–50 μM of EGCG for 12–144 h caused a concentration- and time-dependent reversal of hypermethylated *p16^INK4a^*, retinoic acid receptor ß (*RAR*ß), *MGMT*, and human *mutL* homologue 1 (*hMLH1*) genes.

It was also reported that in an epidemiological study conducted among 73 patients with gastric carcinoma, an increased intake of green tea was significantly associated with the *Cdx2* methylation frequency (*P* = 0.02). The caudal-related homeobox transcription factor (*Cdx2*) is a tumor suppressor gene and frequently inactivated by methylation of its promoter in gastric carcinoma and colorectal cancer cells. Green tea could decrease the *Cdx2* methylation frequency in a dose-dependent manner by 60%, 61%, 75%, and 100% in patients who consumed three or less, four to six, seven to nine and ten cups or more a day, respectively [24].

#### 2.1.3. Antioxidative Effect of Green Tea and (-)-EGCG and Cancer Prevention

In the natural process of oxidation, human bodies produce free radicals. These molecules can cause damage to proteins, lipids and DNA, but are generally cleaned up by substances called antioxidants and systems of antioxidant enzymes before they can insult cells. Several human diseases have a strong association with the oxidative damage in tissues, such as cancer, heart disease, diabetes, Alzheimer's disease, and aging [25, 26]. The term antioxidant originally referred specifically to a chemical that prevented the consumption of molecular oxygen. Green tea is an important antioxidant in the diet. It has been shown that many of the antiproliferative effects of (-)-EGCG are attributable to its antioxidant properties [27]. Rah *et al*. [28] investigated the potential protective roles of green tea polyphenols (GTP) against the injurious effects of reactive oxygen species in human microvascular endothelial cells (HUMVECs). They found that the H_2_O_2_-induced alterations were completely prevented by pre-incubating the endothelial cells with 10 μg/ml GTP for 1 h. When the oxidative stress was induced by xanthine oxidase (XO), cell viability and morphology were also significantly maintained at the same GTP concentration. These results demonstrate that GTP can act as a biological antioxidant in a cell culture experimental model and prevent oxidative stress-induced cytotoxicity in the endothelial cells.

Coimbra *et al*. [29] reported the effect of green tea in protecting the human body from oxidative stress diseases. In 34 human subjects, they evaluated the total antioxidant status (TAS), the two markers of lipid peroxidation products—malonyldialdehyde (MDA) and malonyldialdehyde+4-hydroxy-2(E)-nonenal (MDA+4-HNE)—and the two markers of oxidative changes in erythrocyte membrane, called membrane bound haemoglobin (MBH) and band 3 (a transmembrane protein on erythrocyte) profile. After drinking green tea (1 liter of green tea daily for 4 weeks), they found a significant reduction in serum levels of MDA (by 30.37%) and MDA+4-HNE (by 39.10%) and in the oxidative stress within the erythrocyte, as measured by a significantly lower value of MBH (24.69%) and by changes in band 3 profile towards a normal mean profile [29]. In another *in vivo* study, the total antioxidant capacity of plasma in 10 healthy people was measured at baseline, 60 min and 120 min after ingestion of green tea. The results showed that the total antioxidant capacity of plasma increased by 1.1% at 60 min and 2.1% at 120 min over baseline value in subjects consuming 150 ml of green tea, which was statistically not significant. However, the total antioxidant capacity of plasma after consuming 300 ml of green tea showed a significant increase of 7.0% after 60 min, and 6.2% after 120 min (*P*<0.0001). After consuming 450 ml of green tea, there was an increase to 12.0% after 60 min, and 12.7% after 120 min over baseline value (*P*<0.0001) [30].

### 2.2. Dietary Flavonoids

Flavonoids belong to a subgroups of polyphenols and are widely distributed in the plant kingdom [31, 32]. Flavonoids constitute a large family of compounds including flavanols, flavones, flavonols, flavanones, anthocyanidins, proanthocyanidins and isoflavones [33]. The major sources of flavonoids are from dietary fruit and vegetables. It has been showed that flavonoids possess various biological functions including anti-inflammations, antioxidants and cancer prevention activities [2–5].

#### 2.2.1. Genistein

Genistein is an isoflavone compound and found in soy bean and related products such as Tofu, soy milk and soy sauce [34]. Genistein has been shown to inhibit tumor growth in mouse models of breast, prostate and skin cancers [35, 36]. It has been reported that genistein may protect against spontaneously developing prostate tumors in the transgenic adenocarcinoma of mouse prostate (TRAMP) model. TRAMP mice who were fed a 250 mg/kg diet of genistein significantly down-regulated cell proliferation, EGFR, IGF-1R, ERK-1 and ERK-2 in prostates of TRAMP mice [37].

Treatment with genistein (20 μmol/L) inhibited cell proliferation *in vitro* by approximately 50% in estrogen-independent human breast cancer MDA-MB-231 cells. But in an *in vivo* study, genistein (750 mg/kg AIN-93G diet), fed 3 d before the same cells were implanted into mice, did not significantly inhibit tumor formation or growth [38]. In another study of breast cancer mouse models, treatment of MCF-7 (estrogen-receptor positive) or MDA-MB-468 (estrogen-receptor negative) cell line with genistein before implantation into nude mice diminished tumorigenic potential of these cells [39].

In a skin cancer mouse model study, topic application with genistein was shown to reduce tumor incidence and multiplicity in DMBA-initiated and TPA-promoted skin tumors on SENCAR mouse model by approximately 20 and 50%, respectively. The proposed mechanisms were probably through blockage of DNA adduct formation and inhibition of oxidative and inflammatory events *in vivo* [40]. Mice pretreated with genistein for 2 weeks by gavages had a decreased susceptibility toward DMBA-mediated carcinogenesis on the skin and different organs, associated with increased activity of natural killer cells and increased cytotoxic T lymphocyte activity [41].

#### 2.2.2. Apigenin, Luteolin, Quercetin and Kaempferol

Apigenin and luteolin belong to flavones, and quercetin and kaempferol are flavonols compounds. All of them commonly found in a variety of vegetables: celery, broccoli, onions, peppers, and parsley [33, 42]. These dietary flavonoids have been shown to induce apoptotic cell death in human leukemia, Jurkat T cells, *via* inhibition of proteasome activity [43, 44]. It was found that the order of inhibitory potency against proteasome and potency of inducing apoptosis in Jurkat T cells was apigenin ≥luteolin>quercetin>kaempferol. Through analysis of nucleophilic susceptibility in computer modeling, it was shown that a carbon at position 4 (C_4_) in C ring of flavonoids was an active atom with highest nucleophilic susceptibility to interact with target proteins [44]. By analysis of the structure-activity relationship, we found that deletion of hydroxyl group at the C_3_ position would dramatically increase the potency of flavonoids to inhibit proteasome activity and induce apoptosis in malignant cells [44]. This finding will help researchers synthesize more potent compounds based on structure-activity relationships of natural compounds.

Apigenin was also shown to inhibit proteasome activity and induce apoptosis in human breast cancer MDA-MB-231 cells [45]. Treatment of nude mice bearing human breast cancer MDA-MB-231 xenografts, with 25 or 50 mg/kg of apigenin for 29 days, showed 22% and 43% tumor growth inhibition, respectively, associated with inhibitory proteasome activity and induction of apoptosis [45]. Pretreatment with apigenin (20 and 50 μg/mouse/d) for 2 weeks followed by human prostate cancer 22Rv1 cells implantation in nude mice, tumor volumes were reduced by 39% and 53%, respectively. In another PC-3 tumor model, treatment with apigenin resulted in 32% and 51% inhibition in tumor growth [46]. The proposed mechanism of anti-tumor activity by apigenin was upregulation of WAF1/p21, KIP1/p27, INK4a/p16, and down-modulation of the protein expression of cyclins D1, D2, E and cyclin-dependent kinases (cdk) [46].

### 2.3. Curcumin

Curcumin (diferuloylmethane), a polyphenol compound found in both turmeric and curry powders, is known for its anticancer, antioxidant and anti-inflammatory activities [47–49]. Curcumin has been shown to inhibit the growth of transformed cells and to have a number of potential molecular targets. Curcumin has been shown to inhibit NF-κB and IκB-α kinase (IKK), leading to suppression of proliferation and apoptosis in cell lines of head and neck squamous cell carcinoma [50]. Curcumin has also been shown to suppress fibroblast growth factor-2 (FGF-2) induced angiogenesis through inhibition of expression of matrix metalloproteases (MMPs) in cultured corneal cells [51].

In animal model studies, treatment of hepatocellular carcinoma HepG2 cell-implanted nude mice with curcumin orally, inhibited tumor angiogenesis by measurement of tumor neocapillary density. Through analysis of related angiogenic biomarkers, it was found that expression of cycloxygenase (COX)-2 and serum level of vascular endothelial growth factor (VEGF) were significantly decreased in the curcumin-treated group [52]. In nude mice models, implanted with head and neck squamous cell carcinoma CAL 27 cells, treatment with curcumin inhibited tumor growth. The proposed mechanism was suppression of expression of NF-κB and cyclin D1 [53].

Curcumin appears to be stable at acidic pH, but unstable in neutral and basic pH [54, 55]. In contrast, tetrahydrocurcumin, one of curcumin'smajor metabolites, is quite stable at neutral and basic pH [56]. It was found that curcumin was more potent than tetrahydrocurcumin to inhibit cell proliferation in cultured HepG2 cells. However, in HepG2 implanted nude mice, treatment with tetrahydrocurcumin resulted in more potent inhibition against angiogenesis than curcumin [57].

In a phase I clinical trial, curcumin was taken orally for 3 months for cancer patients. The serum concentration of curcumin peaked at 1 to 2 hours. The average peak serum concentrations after taking 4, 6 and 8 g of curcumin, were 0.51, 0.63 and 1.77 μmol/L, respectively [58]. This study demonstrated that curcumin was not toxic to humans up to 8 g/day when taken orally for 3 months [58].

A phase II trial of curcumin in patients with advanced pancreatic cancer evaluated the toxicity and activity of curcumin. Patients were treated with 8 g of curcumin daily by mouth for two months. Eleven patients were evaluated for response, and 15 were evaluated for toxicity. The results suggest that curcumin is well tolerated and no toxicities have been observed. Four patients have stable disease for two to seven months, and one patient had a brief partial remission indicated by 73% reduction in tumor size, by Response Evaluation Criteria In Solid Tumors (RECIST) for one month [59]. More clinical trials are needed to evaluate its biologic activities and molecular targets in cancer patients.

It should mention that in clinical trials of oral administration of curcumin to human cancer patients, the systemic availability and blood level of curcumin was found to be negligible, due to poor absorption of this compound [60, 61]. Therefore scientists have been developing higher bioavailability and more potent anticancer compounds through modifying and synthesizing analogues of curcumin. Adams *et al*. reported that several synthesized curcumin analogs inhibited tumor cell growth with a higher potency than the commonly used chemotherapeutic drug, cisplatin, and one of the analogues was equal potent as the anti-angiogenic drug TNP-470 [62]. Another research group [63] synthesized more than 50 curcumin analogs through α, β-unsaturated ketone modification. Amount these analogs, three of them (named by authors as GO-Y016, GO-Y030 and GO-Y031) showed >30 times greater potency than natural curcumin for their cell growth-inhibitory activity in human colon cancer HCT116 cells [63]. The possible mechanisms include decreased expression levels of oncoproteins, β-catenin, Ki-ras, cyclin D1, and ErbB-2, at concentrations much lower than those normally used for curcumin [63].

## 3. Conclusion

Natural compounds have been extensively studied and have shown anti-carcinogenic activities by interfering with the initiation, development and progression of cancer through the modulation of various mechanisms including cellular proliferation, differentiation, apoptosis, angiogenesis, and metastasis. However, further investigations are needed, especially focusing on molecular targets, mechanism-based animal and clinical studies to fully realize their potential usages and biological activities. Additionally, biological activities of these natural compounds are generally not potent enough and higher concentrations would be required to achieve the expected biological effects. Furthermore there are bioavailability and stability issues associated with some natural compounds. Therefore, based on chemical structures of natural compounds to synthesize more analogical compounds with greater potency and more stable properties is another important topic for investigation. By comparison of the structure-activity relationship (SAR) between natural and synthetic compounds, scientists have developed a series of novel analog compounds with improved bioavailability and potency of antitumor activity, compared with the natural parent compounds. These synthetic compounds include a Pro-drug of EGCG synthesized in our laboratories [64] and curcumin analogs such as GO-Y016, GO-Y030 and GO-Y031 [63]. These successful examples will encourage researchers to synthesize, screen and discover more and better natural compound analogs that will eventually benefit cancer patients in the clinic.
